# Online assessment of patients' views on hospital performances using Rasch model's KIDMAP diagram

**DOI:** 10.1186/1472-6963-9-135

**Published:** 2009-07-31

**Authors:** Tsair-Wei Chien, Wen-Chung Wang, Hsien-Yi Wang, Hung-Jung Lin

**Affiliations:** 1 Department of management, Chi-Mei Medical Center, Taiwan, Republic of China; 2 Department of Hospital and Health Care Administration, Chia-Nan University of Pharmacy and Science, Tainan, Taiwan, Republic of China; 3 Assessment Research Center, Hong Kong Institute of Education, Tai Po, Hong Kong; 4 Department of Nephrology, Chi Mei Medical Center, Taiwan, Republic of China; 5 Department of Emergency Medicine, Chi-Mei Medical Center, Taiwan, Republic of China; 6 Department of Biotechnology, Southern Taiwan University of Technology, Taiwan, Republic of China

## Abstract

**Background:**

To overcome the drawback of individual item-by-item box plots of disclosure for patient views on healthcare service quality, we propose to inspect interrelationships among items that measure a common entity. A visual diagram on the Internet is developed to provide thorough information for hospitals.

**Methods:**

We used the Rasch rating scale model to analyze the 2003 English inpatient questionnaire data regarding patient satisfactory perception, which were collected from 169 hospitals, examined model-data fit, and developed a KIDMAP diagram on the Internet depicting the satisfaction level of each hospital and investigating aberrant responses with Z-scores and MNSQ statistics for individual hospitals. Differential item functioning (DIF) analysis was conducted to verify construct equivalence across types of hospitals.

**Results:**

18 of the 45 items fit to the model's expectations, indicating they jointly defined a common construct and an equal-interval logit scale was achieved. The most difficult aspect for hospitals to earn inpatients' satisfaction were item 29 (staff told you about any medication side effects to watch when going home). No DIF in the 18-item questionnaire was found between types of hospitals, indicating the questionnaire measured the same construct across hospitals. Different types of hospitals obtained different levels of satisfaction. The KIDMAP on the Internet provided more interpretable and visualized message than traditional item-by-item box plots of disclosure.

**Conclusion:**

After removing misfit items, we find that the 18-item questionnaire measures the same construct across types of hospitals. The KIDMAP on the Internet provides an exemplary comparison in quality of healthcare. Rasch analysis allows intra- and inter-hospital performances to be compared easily and reliably with each other on the Internet.

## Background

Many studies have discussed patients' perception about their hospitals and the benefit of listening to other patients' experience when choosing a hospital [1-8]. There has been a rapid increase in websites that allow patients to rate their hospitals [9,10]. In recent years almost all healthcare providers have been explicitly required to conduct surveys of their patients' healthcare experience. Data from such surveys have been published in journals or on websites (Leapfrog Group [11] & Patient Opinion [12]) but they rarely comply with the Web 2.0 requirement to improve communication between people via social-networking technologies [13]. Those surveys often use individual item-by-item box plots to disclose patient views on hospital service quality. They are thus unable to provide hospital staff with aberrant responses for prudence and further improvement in performance of patient-centered satisfaction, nor to help patients choose hospitals according to an overall performance level.

### Critiques of traditional Likert-type patient satisfaction surveys

Web 2.0 has changed the relationship between patients and hospitals [4,14]. Critiques of traditional patient satisfaction surveys [7,15] have led to a new emphasis on measuring patients' experiences rather than their satisfaction levels only [16]. The England Picker Institute Europe (or EPIE for short [17]) has created such questionnaires (shown in Table 1) and ask patients to report in detail their experience with a particular provider at a specific point of time by answering questions about whether or not certain processes or events occurred during the course of a specific episode of care [18], rather than just ask patients to rate their care on a Likert-type scale [19].

**Table 1:** Picker's 45-item inpatient questionnaire

Category & Items	
***Admission to hospital***	
1	Was your hospital stay planned in advance or an emergency?
2	How organized was the care you received in A & E (or the admissions unit)?
3	Following arrival at the hospital, how long did you wait before admission to a room or ward and bed?
4	How do you feel about the length of time you were on the waiting list before your admission to hospital?
5	Were you given enough notice of your date of admission?
6	Were you given a choice of admission dates?
7	Was your admission date changed by the hospital?
8	Were you given a choice about which hospital you were admitted to?
9	You feel wait a long time to get to a bed on a ward?
***The Hospital and Ward***	
10	During your stay in hospital, did you ever share a room or bay with patients of the opposite sex?
11	Ever bothered by noise at night from other patients?
12	Bothered by noise at night form hospital staff?
13	How clean was the hospital room or ward?
14	How clean were the toilets and bathrooms?
15	How would you rate the hospital food?
***Doctors***	
16	Did you get answers that you could understand form a doctor?
17	Having confidence and trust in the doctors treating you?
18	Doctors talked in front of you as if you weren't there?
***Nurses***	
19	You get answers that you could understand from a nurse?
20	You had confidence and trust in the nurses treating you?
21	Nurses talked in front of you as if you weren't there?
22	Were there enough nurses on duty to care for you in hospital?
23	Staff saying one thing and another quite different happened to you?
24	Were you involved to be in decisions about your care and treatment?
25	How much information about condition or treatment was given to you?
26	Your family talk to a doctor had enough opportunity to do so?
27	Hospital staffs talk about your worries and fears?
28	Given enough privacy when discussing your condition or treatment?
27	Did you find someone on the hospital staff to talk to about your worries and fears?
30	How long after using call button before you got the help you needed?
29	Were you given enough privacy when being examined or treated?
32	Were your scheduled tests, x-rays or scans performed on time?
***Pain***	
33	Were you ever in any pain?
34	Hospital staff did everything the could to help you control your pain?
Leaving Hospital	
35	On the day you left hospital, was your discharge delayed for any reason?
36	What was the main reason for the delay?
37	How long was the delay?
38	Staff explained the purpose of the medicines you could understand?
39	Staff told you about any medication side effects to watch when going home?
40	Staff told you about any danger signals you should watch for after going home?
41	Doctors or nurses gave your family all the information they needed to help you?
42	Staff told you how to contact if worries happened after leaving?
***Overall***	
43	Overall, did you feel you were treated with respect and dignity while you were in hospital?
44	How would you rate well the doctors and nurses worked together?
45	Overall, how would you rate the care you received?

### Inappropriate individual item-by-item box plots of disclosure

**Figure 1:**
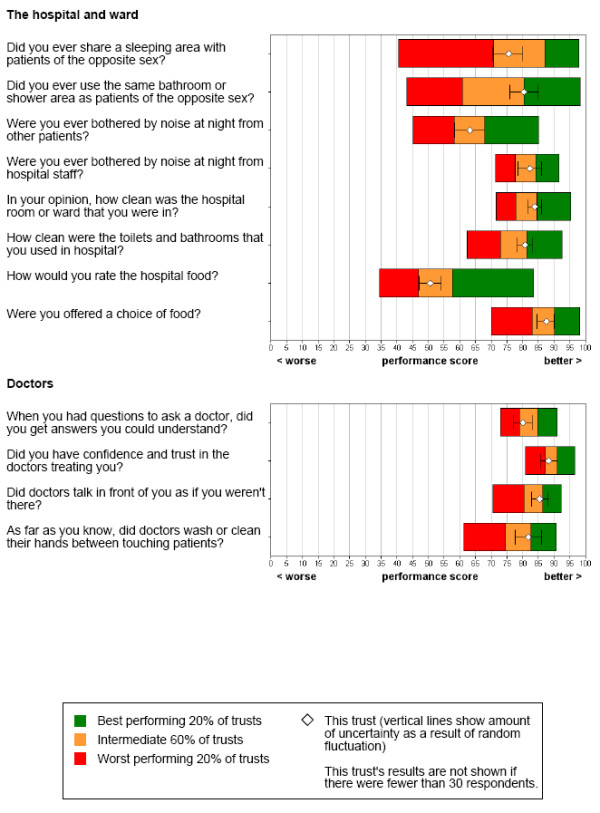
**Item-by-item disclosures by the box plots**. (Retrieved from ).

Items in Picker's questionnaire are often analyzed and presented individually, one item at a time (e.g., item-by-item box plots of disclosure as shown in Figure 1). In so doing, global interrelationships between items are invisible [20]. Besides, measurement error in a single item is often very substantial but it is ignored in such an item-by-item analysis. We are therefore concerned with the interrelationship between items when they are, in effect, measuring a single construct. Advanced analysis is required.

Item response theory (IRT) or Rasch measurement [21] provides such an advanced analysis to take into account the interrelationship among items. A newly designed diagram can be provided to accurately report patients' experiences for each hospital.

### Objective measurement requirements

There are three major problems in conventional analyses of Picker's questionnaires: (a) *interval scores*: Raw scores of Picker's questionnaires are ordinal but have been mistakenly treated as interval and analyzed with linear factor analysis; (b) *graphic diagrams with correlated items: *more efforts should be made to provide meaningful and simple diagrams (e.g., Google Image Labeler labels digital photographs according to their content) by making the task a simple game in which contestants (i.e., hospitals in this study) must both collaborate and compete with each other; and (c) *aggregate scores*: the benchmark reports that present item-by-item results for patients to compare with national results not only fail to help patients understand the overall performance of each hospital with an aggregate score, but also lack an objective metric to quantify the difference between hospitals. Details are described as follows:

### (1) Interval and additive scores for comparison

How to measure individual differences on an interval scale [22-24] is an important issue in health care systems. The United Kingdom [25] has introduced a pay-for-performance contract for family practitioners according to performance with respect to 146 quality indicators covering clinical care for 10 chronic diseases, organization of care, and patient experience. In this contract, interval scores should be ensured beforehand such that they can be meaningfully compared with each practitioner. Stout [26] quoted Lord and Novick [27] as saying, "A major problem of mental test theory is to determine a good interval scaling to impose when the supporting theory implies only ordinal properties".

### (2) KIDMAP improvement of communication on the Internet

KIDMAP (also called Diagnostic Map), derived from Rasch measurement, provides an output for each individual and summarizes expected and unexpected response patterns. The first large-scale implementation of KIDMAP took place in the Los Angeles Independent County School District in the early 1980s. Hundreds of thousands of KIDMAP reports have been distributed to parents [28,29]. In this study, we implement KIDMAP to summarize patients' perception about each individual hospital's performances, distribute KIDMAP reports to hospital staff through the Internet, so that they can easily recognize key indicators for improvement. The reports also allow patients to compare hospitals' performance in quality of healthcare.

Quest [30] is the first software that produces KIDMAP reports. Chien et al. [31] developed a web-based KIDMAP (shortly Web-KIDMAP) on hospital indicator management, and they argued that Web-KIDMAP empowers diagnostic information by placing responses in four quadrants, not only for hospitals to improve their performances, but also for patients to better choose hospitals.

### (3) Aggregate scores produce useful results for questionnaires

The EPIE questionnaires require patients to report what happened to them rather than how satisfied they are. EPIE believes that questionnaires about satisfaction do not produce very useful results. A better approach is to require patients to report their experiences in hospitals so as to identify hospital strength and weakness, which allows hospitals to target problems and improve services [32]. Accordingly, an aggregate score for satisfaction level of each hospital is essential and a quality control fit statistic indicator [33] is needed so that hospitals' performance can be quantified and compared.

### Aims of the study

In this study, we apply Rasch analysis to (a) examine whether items in the EPIE questionnaire fit the model's expectations so that the underlying latent trait (henceforth referred to as satisfaction level) can be quantified and an interval scale can be obtained; (b) display the KIDMAP diagram [28,29] on the web to help patients understand the overall performance of individual hospitals; and (c) quantify the performances among hospitals along a continuum single construct.

## Methods

### Instrument and data collections

With permission, we downloaded datasheets from a total of 169 hospitals in the 2003 English inpatient questionnaire from EPIE [17]. The original 45-indicator questions (Table 1) have been scored using a scale of 0 to 100. The scores indicate the extent to which the patient's experience could have been improved. A score of 0 is assigned to all responses that reflect considerable need for improvement, whereas an answer option that has been assigned a score of 100 refers to a positive patient experience.

The questionnaire survey was carried out in all acute and specialist National Health Service (NHS) trusts (i.e., hospitals) in England that care for adult inpatients. Each of the 169 trusts, shown in Table 2, identified a list of 850 randomized eligible patients who had been discharged from the trust counting back from the last date of September, October or November 2003. The questionnaires were sent to 143,322 patients (≅ 850 × 169) and 88,308 completed questionnaires were returned. Patients were eligible to participate if they had had at least one overnight stay, were over 18 years old and were not maternity or psychiatry patients. Among the eligible patients, 54% were women, 12% were aged 16 to 35 years, 17% were aged 36 to 50 years, 27% were aged 51 to 65 years, 33% were aged 66 to 80 years and 12% were aged 81 or over. The original data for the 88,308 patients were not available to us. Instead, the aggregated data for the 169 hospitals were shown on EPIE website for download and then analyzed by the authors.

**Table 2:** Types of the 169 Hospitals in this study

Type	Type	Quantity
Small	Small acute outside London	27
Small	Small acute London	4
Medium	Medium acute outside London	38
Medium	Medium acute London	9
Large	Large acute outside London	39
Large	Large acute London	4
Specialty	Acute specialist	12
Specialty	Orthopaedic	4
Teaching	Acute teaching outside London	16
Teaching	Acute teaching London	9
Large	Multi-service	7

Total		169

### Data transformation and Rasch analysis

#### 1. Data transformation

We transformed the 45 items from a raw score of 0–100 into five ordinal categories by a logarithm function of those original response data (i.e., integer log(original response raw score): 20, 60 and 100 to be 3, 4 and 5, respectively). From these five categories, the Rasch rating scale model [34] was fitted using the computer software WINSTEPS [35] with maximum likelihood estimation technique to yield a respective interval logit scaled score for individual hospitals [see Additional file 1], which is different from the traditional summed scores in ordinal nature as their satisfactory levels.

#### 2. Rasch analysis

Rasch models [21] are latent trait models which imposes a probabilistic relationship between the level of latent trait (referred to as satisfaction level for a hospital in this study) and the items used for measurement (referred to as item difficulty). A fundamental assumption underlying Rasch models is unidimensionality, which can be assessed through point biserial correlation, Rasch fit indicators and Rasch factor analysis [36-38].

Rasch fit indicators include non-weighted (outfit) and weighted (infit) mean square errors (MNSQ) for items. The outfit MNSQ directly squares and averages standardized residuals; whereas the infit MNSQ averages standardized residuals with weights [39,40]. When items meet the model's expectations, their outfit or infit MNSQ will have an expected value of 1. In contrast, an MNSQ very close to zero indicates redundancy of items and an MNSQ far lager than 1 indicates too much random noise.

For rating scales a range of 0.5 to 1.5 for the MNSQ statistics is often recommended as the critical range for a productive of measurement [41-43]. Items with an iutfit or infit MNSQ beyond this range are regarded as misfit. Successive Rasch analyses should be performed until all items satisfied the model fit requirements. Rasch model is superior to factor analysis in terms of confirming a factor structure [44]. When misfit items are identified and removed, unidimensionality is guaranteed and interval measures can be produced [45].

Principal components analyses (PCA) of the residuals from Rasch analysis can also be used to check the assumption of unidimensionality [46,47]. The following criteria are recommended to determine whether the assumption of unidimensionality holds: (a) the variance explained by the Rasch factor (the underlying construct) should be 4 times greater than that of the first principal component in the residuals; (b) the variance explained by the Rasch factor should be greater than 50%; (c) the eigenvalues of the residuals should be smaller than 3; and (d) the percentage variance explained by the first principal component in the residuals should be less than 5% [41,48].

### Assessment of differential item functioning (DIF)

To make comparison across different groups of hospitals, the test construct must remain invariant across groups. DIF analysis is a means to verify construct equivalence over groups [49]. If construct equivalence does not hold over groups, meaning that different groups respond to individual questions differently after holding their latent trait levels constant, then the estimated measures could not be compared directly over groups. In this study hospital type (small – large acute, teaching, etc) were examined for DIF. A *p*-value less than .05 indicates a DIF.

### KIDMAP shown on the web

In order to improve the traditional KIDMAP profile, we developed an innovative web-based KIDMAP, called Web-KIDMAP, which reveals valuable information for hospital staff and patients in real time. Together with infit and outfit MNSQ, Web-KIDMAP reveals the strength and weakness of each hospital.

## Results

### Examining a single underlying construct

Among the 45 items, a total of 18 items (shown in Table 3) met the model's expectations fairly well (infit and outfit MNSQ within 0.5 and 1.5). These 18 items also covered those seven specified categories of Picker's original inpatient questionnaire. The 18 items had point-biserial correlations in a range from 0.66 to 0.84. A principal component analysis on the residuals of Rasch scores showed no additional factors in that (a) the variance explained by items was more than 4 times greater than that of the first principal component (i.e. dividing 68% by 4.6% yields 14.78 times); (b) the variance explained by the Rasch factor was 68%, greater than that the cutoff of 50% (c) the first eigenvalue was 2.6, less than the cutoff of 3; and (d) the percentage of variance explained by the first principal component was of 4.6%, less than 5%. These results indicated that there was a good model-data fit and that the assumption of unidimensionality held for these 18 polytomous items. The category Rasch-Andrich thresholds (step difficulties) were ordered as -3.76, -1.91, 1.57 and 4.11.

**Table 3:** Item difficulties with DIF-free and Fit MNSQ statistics of the 15 items in the 2003 English inpatient questionnaire of the Picker Institute Europe

No.	Category & Item	Difficulty		MNSQ			DIF-free
		Logit	*SE*	Infit	Outfit	PTME^§^	p-value*
	***Admission to hospital***						
2	How organized was the care you received in A&E?	-0.95	0.15	1.05	1.18	0.7	.736
5	Were you given enough notice of your date of admission?	-1.08	0.15	1.02	0.94	0.69	.968
9	You feel wait a long time to get to a bed on a ward?	-0.63	0.14	1.2	1.1	0.74	.116
	***The Hospital and Ward***						
11	Ever bothered by noise at night from other patients?	1.58	0.13	1.50	1.50	0.74	.289
12	Bothered by noise at night form hospital staff?	-1.1	0.15	1.02	1.02	0.69	.287
	***Doctors***						
17	Having confidence and trust in the doctors treating you?	-1.1	0.15	0.61	0.66	0.79	.553
18	Doctors talked in front of you as if you weren't there?	-1.12	0.15	1.12	1.07	0.67	.120
	***Nurses***						
19	You get answers that you could understand from a nurse?	-1.12	0.15	0.56	0.57	0.8	.114
23	Staff saying one thing and another quite different happened to you?	-1.1	0.15	0.98	0.89	0.71	.199
24	Were you involved to be in decisions about your care and treatment?	0.67	0.14	0.77	0.77	0.84	.068
27	Hospital staff talk about your worries and fears?	2.22	0.13	0.56	0.52	0.81	.364
30	How long after using call button before you got the help you needed?	0.42	0.14	0.91	1.21	0.66	.253
	***Pain***						
34	Hospital staff did everything they could to help you control your pain?	-1.12	0.15	0.82	0.87	0.74	.906
	***Leaving Hospital***						
38	Staff explained the purpose of the medicines you could understand?	-1.1	0.15	0.97	0.87	0.71	.972
39	Staff told you about medication side effects when going home?	3.78	0.13	1.22	1.16	0.81	.407
41	Doctors or nurses gave your family information needed to help you?	2.76	0.13	1.37	1.44	0.78	.090
42	Staff told you how to contact if worries happened after leaving?	-0.3	0.14	1.33	1.19	0.73	.344
	***Overall***						
44	How would you rate well the doctors and nurses worked together?	-0.71	0.14	0.75	0.67	0.8	.303

	Mean	0.00	0.14	0.99	0.98	0.75	
	*SD*	1.56	0.01	0.28	0.28	0.05	

Note. *DIF CHI-square probability from TABLE 30.4 of WINSTEPS software

^§^biserial correlations

Overall, these 18 items exhibited a good model-data fit. Hence, they measured a single construct for patient satisfaction and an interval scale of logits was achieved for further comparison and analysis [38]. The hospital measures ranged from -1.59 to 9.63 with mean 2.64 and standard deviation 2.09, indicating items were easier for these hospitals and a wide range of hospitals dispersed on the interval scale.

The hospital sample separation reliability was 0.94 (Cronbach's α [50] = 0.96), indicating that these 18 items could differentiate the hospitals very well. The separation index for the items (a measure of the spread of the estimates relative to their precision) was as high as 4.01, allowing us to differentiate between five statistically distinct strata of item difficulties with the formula of strata = (4 × 4.01 + 1)/3 [51].

Analysis of variance on the hospital measures reveals a significant difference (*F *= 8.318; *p *< .001) among types of hospitals: General practices (*M *= 4.72 logits) performed the best, followed by Large hospitals (*M *= 3.47 logits), Teaching hospitals (*M *= 2.62 logits), Small hospitals (*M *= 2.25 logits), and Medium hospitals (*M *= .82 logits).

### Item properties

The three most difficult items to be satisfied by patients were: item 39 (Staff told you about any medication side effects to watch when going home), item 41 (Doctors or nurses gave your family information needed to help you) and item 27 (Hospital staff talks about your worries and fears). The easiest one was item 34 (Hospital staff did everything they could to help you control your pain). The mean and standard deviation of items was 0.00 and 1.52, respectively. All of items were in a range of absolute 4.9 logits. The item difficulties were well spread out across the hospitals, indicating that these items could differentiate hospitals fairly well so as to reach a hospital separation reliability of 0.94.

Item invariance refers to the fact that the estimated item location parameters should not depend on the sample used to calibrate the estimates [50]. Table 3 shows that no DIF items were found across different types of hospitals, suggesting these items measure the same construct across types of hospitals such that their performances can be directly compared.

### KIDMAP used for diagnosing hospitals

**Figure 2:**
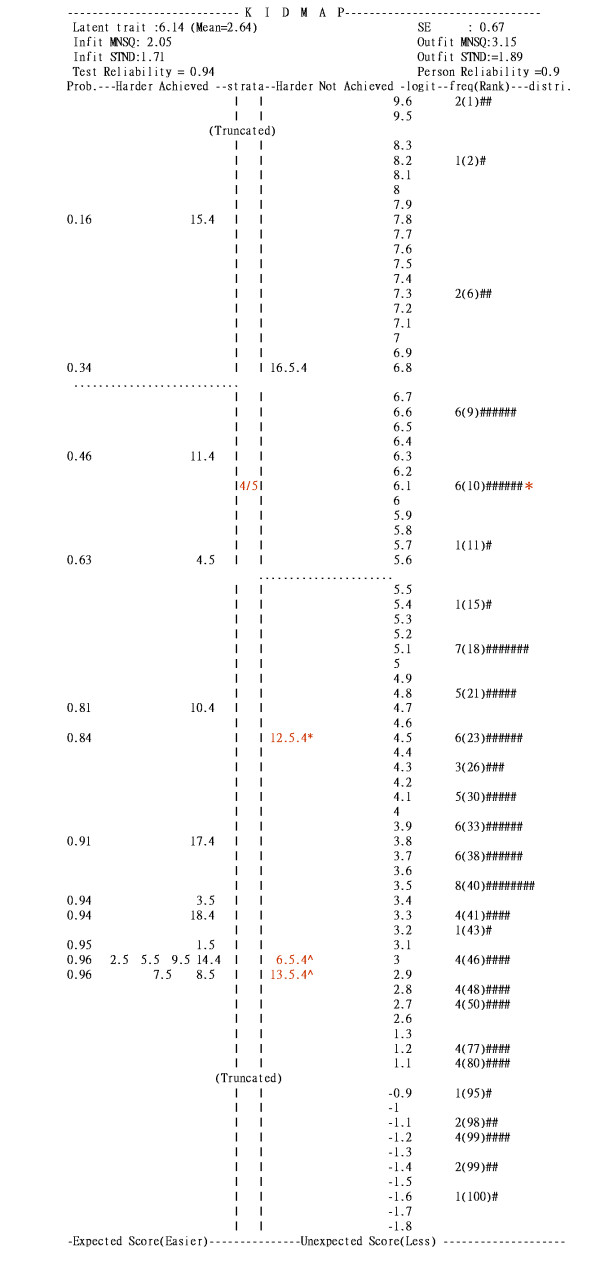
**KIDMAP profile of an actual assessed hospital**.

Figure 2 shows a Web-KIDMAP for a particular hospital. In the right-hand bottom corner (the 4th quadrant), there are six 'easier not achieved' items that the hospital was expected to have achieved given the performance estimate of 6.14 logits and the percentile rank of 10 (see to the right of the percentile column in Figure 2).

The most unexpected errors among the three items, one noted with an asterisk (*) and two with a caret (^), are indicated as statistically significantly different (*p *< 0.05 and *p *< 0.01, respectively). The label 6.5.4 in the 4th quadrant means that the 5^th ^category of item 6 was endorsed as 4 by the hospital. Actually, the hospital (6.41 logits) had a very good chance to achieve a score higher than 4 but failed to achieve (e.g., see the left-hand side in Figure 2). These unexpected responses are informative and worth noting, because the hospital's weakness did not match the patients' perception. Note that the unexpected response was identified by inspecting the hospital's own performance level (i.e., self comparison), rather than the averaged scores in Picker's item-by-item diagram, Figure 1.

### Three steps to read the Web-KIDMAP

We analyzed the data from the 2003 EPIE inpatient questionnaire [17] and developed a Web-KIDMAP diagram that could be visualized on the Internet for (a) inter-hospital comparison (by inspecting performance levels along the logit scale), (b) intra-hospital comparison (by inspecting response patterns and residual Z-scores), and (c) model-data fit checking (by inspecting MNSQ statistics).

## Discussion

### Findings

We use Rasch measurement to construct an interval logic scale for patient satisfaction on hospital performance, utilize fit statistics to detect aberrant response pattern, and develop a visual representation on website, Web-KIDMAP, to overcome the drawback in traditional individual item-by-item box plots of disclosure for patient views on hospital service quality.

The item-by-item analysis of the Picker's questionnaire ignores measurement error that is embedded in an item. In reality, a self-report item (like those in Picker's questionnaire) is very imprecise and contains a large measurement error, which invalidates item-by-item analysis. Besides measurement error, item-by-item analysis is useful only when each provides feedback information about an individual practice (or program, treatment, etc.). It is hard to believe that there are 45 kinds of practice that have been routinely carried out in a hospital, such that we need 45 items to provide feedback information, one item for one kind of practice.

Rasch analysis considers measurement error in an item and yields global and local feedback information for hospital performance (e.g., item and person fit analysis, DIF analysis, item difficulty hierarchy, the Wright map, the KIDMAP, etc.).

### Strengths of the study

#### 1. Objective measurement by Item-fitting with the Rasch model

Using the Rasch approach, we have examined patient experience from the EPIE 2003 inpatient questionnaire. Of the original 45 items, 18 items meet the model's expectations fairly well so that the underlying satisfaction latent trait is successfully quantified via the 18 items. It turns out that the most difficult area for hospitals to earn inpatients' satisfaction is "Staff told you about any medication side effects to watch when going home."

#### 2. KIDMAP based on interval logit scores yielded by Rasch analysis

KIDMAP is derived from performance diagnosis of students in education fields [28,29]. It is based on an interval scale constructed by Rasch analysis. The Web-KIDMAP approach is invented by the authors as a means to perform macro- and micro-examinations of hospital performance [31], to provide much more valuable information than that generated by the traditional Quest software [30]. In this study we show how to construct a visualized program of Web-KIDMAP in order to describe a hospital's overall performance, diagnose its response patterns, and then transplant all the information onto a website.

#### 3. Quality control Item fit statistics

The strength (on the top-left corner in Figure 2) and weakness (on the bottom-right corner in Figure 2) of the hospitals are displayed on Web-KIDMAP to allow hospital staff to generate performance improvement plans and patients to select hospitals. Web-KIDMAP engenders a basis of intra- and inter-hospital comparison in quality of healthcare: considering why the weakness responses are endorsed by patients, what is warranted is further support the strengthened indicators, and what possible tools or methods are called for to satisfy patients' targeted expectations in the top-right corner (the 1st quadrant of Figure 2) on which the performance ability estimate could be upgraded to a level higher than 6.14 logits in Figure 2.

### Limitations of the study

High quality healthcare feedback is important. More work is needed to further enable the administration of an effective feedback system in healthcare settings. In this study, we demonstrate how a visualized Web-KIDMAP can be used to compare patient perception on healthcare service on the Internet. Users may need some background knowledge to interpret Web-KIDMAP properly.

Due to DIF and misfit to the model, 27 items are removed. This is because these items do not work with the other items harmoniously to measure the same construct, not because they are useless or unimportant to hospital performance. Future studies can investigate importance of these 27 items. If they are very important to hospital performance, then a stand-along scale should be developed. Future study can further macro-examine the test properties and to micro-investigate the item response with/without errors or abnormalities [52].

### Applications

**Figure 3:**
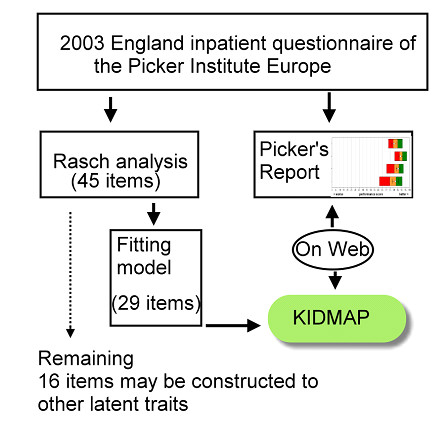
**Flow chart of this study**.

KIDMAP is not designed to replace traditional item-by-item box plots as shown in Figure 1, but to complement them. Furthermore, Web-KIDMAP is highly dynamic and easily interpretable with graphic tools as well as the exemplary comparisons in quality of healthcare, especially on the Internet referring to Figure 3.

Fit MNSQ statistics describe measurement quality. As for the illustration in Figure 2, an outfit MNSQ of 3.15 indicates that the patients' responses to the hospital does not provide useful information and there is too much unexplained noise in the observation (such as those three red colored items in Figure 2)[52].

The indicators in the top-left corner (the 2nd quadrant) are the hospital's strength associated with the category 'harder ones but achieved'. The probability of endorsing such an item by the hospital is shown in the ultimate left column. "4/5", located in the middle of the Web-KIDMAP, indicating the performance estimate on location of 6.14 logits classified as the 4th best stratum (note: the separation index for the persons is as high as 4.01, allowing us to differentiate between five statistically distinct strata of person abilities with the formula of strata (= (4 × 4.01 + 1)/3). Percentile ranks, frequencies and the distribution of the norm-reference from 169 hospitals are innovated and shown on the right-hand side, differing from the traditional KIDMAP provided by the Quest software [30].

### Further studies and suggestions

Web-KIDMAP provides a response profile of an individual hospital. Hospital staff can use Web-KIDMAP to inspect patients' perception about hospital performance. Aberrant responses in those items of "easier not achieved" deserve more attention. The Z-scores in Figure 2 provide an opportunity for a hospital to examine its strength and weakness by self-comparison rather than compared with other hospitals, so as to upgrade the overall performance level.

The transformation of ordinal raw scores into interval logits using Rasch measurement [21.38] within patient-centered research is worth further research. We believe that Web-KIDMAP [31] is indicative, efficient and effective and can easily facilitate patient-centered clinical environment.

We herein propose that healthcare informatics be regarded as a whole newly integrated academic discipline, one that should be devoted to the exploration of the new possibilities that informatics is creating for both hospital staff and patients in relation to health and healthcare issues [53,54].

These 18 items can be added to a static questionnaire as a daily routine for examining healthcare quality of a hospital or as item banks created for a touch screen version of computer adaptive testing (CAT) to reducing patient burdens in responding questions [55], which studies are worth carrying out in the future.

## Conclusion

The England Picker Institute Europe annually discloses reports of patients' experience with a particular provider at a specific point by an item-by-item approch with box plots indiividually. In this study, we apply IRT-based Rasch analysis to create Web-KIDMAP to help patients understand the overall performance of individual hospitals, and quantify the performances among hospitals along an interval scale. Web-KIDMAP provides an newly developed comparison in quality of healthcare and allows intra- and inter-hospital comparison on the Internet.

## Abbreviations

IRT: item response theory; MNSQ: mean square error; NHS: National Health Services.

## Competing interests

The authors declare that they have no competing interests.

## Authors' contributions

TW designed the study and performed the analysis, interpreted the results, and drafted the manuscript. WC contributed to the revision of manuscript. HY helped to interpret the results and participated in the formulation of the discussion. HJ helped to interpret the results and contributed to the discussion. All authors reviewed and edited the manuscript for intellectual.

## Pre-publication history

The pre-publication history for this paper can be accessed here:



## Supplementary Material

Additional file 1 **The control file of WINSTEPS software**. The data provided represent the Rasch analysis of the 18 items fitting to the model's expectations. Click here for file
